# Gastrodin reduces myocardial ischemia/reperfusion injury via transgelin2/CNPase-mediated apoptosis regulation

**DOI:** 10.3389/fphar.2025.1604408

**Published:** 2025-07-14

**Authors:** Changyan Li, Peng Rao, Xiang Liu, Lin Yang, Yongliang Jiang, Gaosheng Yin, Shuangxiu Li, Ping Yang, Lin Sun

**Affiliations:** ^1^ Yunnan Key Laboratory of Stem Cell and Regenerative Medicine, School of Rehabilitation, Kunming Medical University, Kunming, China; ^2^ Department of Orthopedic Surgery, The First People’s Hospital of Yunnan Province, Kunming, China; ^3^ Department of Cardiology, the Second Affiliated Hospital, Kunming Medical University, Kunming, China

**Keywords:** myocardial ischemia/reperfusion injury, Gastrodin, apoptosis, CNPase, transgelin2

## Abstract

**Background:**

Myocardial ischemia-reperfusion injury (MIRI) frequently occurs during rapid restoration of blood flow in the infarcted myocardium. While Gastrodin (GAS) mitigates MIRI, its mechanism requires further exploration.

**Methods:**

We evaluated GAS effect in SD rats following 45-min left coronary artery ligation and reperfusion. GAS (intraperitoneal) was administered preoperatively for 3 days. Triphenyltetrazolium chloride (TTC) staining was used to detect infarct size. The cardiac function was monitored by the Langendorff isolated cardiac perfusion system. Hematoxylin-Eosin (H&E) staining was applied to detect cardiac injury. H9c2 cells underwent oxygen and glucose deprivation (OGD) and were subsequently restored to normal culture conditions, mimicking MIRI. Cell Counting Kit-8 (CCK-8) was used to detect the cytotoxicity of GAS. Myocardial cell injury was determined by detecting lactate dehydrogenase (LDH) level in the medium. The expression of protein was detected by Western blot (WB) and immunofluorescence (IF) assay. Coimmunocoprecipitation (Co-IP), coupled with molecular docking detected the combination among transgelin2 (TG2), and CNPase.

**Results:**

GAS reduced the size of myocardial infarction, alleviated myocardial fiber damage, and ameliorated MIRI-mediated cardiac dysfunction. Mechanistically, GAS inhibited apoptosis by restoring MIRI-altered TG2/CNPase expression. TG2 directly bound and negatively regulated CNPase. CNPase deficiency enhanced MIRI amelioration by reducing apoptosis.

**Conclusion:**

Taken together, GAS protects against MIRI by modulating apoptosis through the TG2/CNPase pathway, revealing a novel therapeutic target.

## Graphical Abstract

**Figure:**
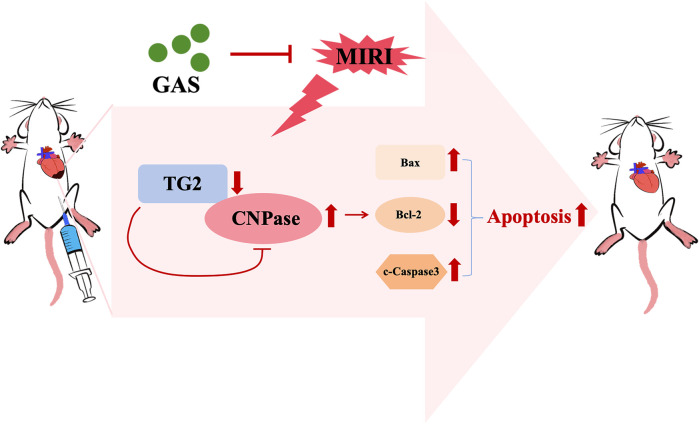
Gastrodin alleviates MIRI by suppressing TG2/CNPase-mediated apoptosis. In MIRI, reduced TG2 expression in cardiomyocytes leads to elevated CNPase levels due to disrupted TG2-CNPase binding and regulatory control. This imbalance promotes cardiomyocyte apoptosis. GAS administration counteracts this pathway by enhancing TG2 expression, thereby normalizing CNPase levels and attenuating apoptotic signaling.

## 1 Introduction

Acute myocardial infarction (AMI) persists as a leading global cause of cardiovascular mortality (Thiele et al., 2020). The preferred treatment for AMI is percutaneous coronary intervention (PCI), as it effectively prevents myocardial necrosis and mitigates myocardial damage through prompt reperfusion and restoration of coronary blood flow (Reed et al., 2017). However, reperfusion benefits are constrained by paradoxical reperfusion injury, also named MIRI, and the post-procedural mortality ranges from 6% to 14% (Wu et al., 2019; Yoneyama et al., 2021). Mitigating MIRI thus constitutes a crucial therapeutic challenge in AMI management (Lisa and Bernardi, 2015; Zhang J. et al., 2019). However, there is a scarcity of safe and efficacious medications for alleviating MIRI (Hausenloy and Yellon, 2016).

Cardiomyocyte death represents a hallmark feature of MIRI (Xiang et al., 2024). Apoptosis, a regulated programmed death pathway (Chen and Lin, 2017), critically contributes to MIRI pathogenesis (Zhang and Herman, 2006). Notably, apoptosis repressor with caspase recruitment domain protects the heart from MIRI by inhibiting key apoptotic signaling cascades (Liu et al., 2023). Traditional Chinese medical compounds constitute established therapeutic options for cardiovascular diseases (Jiang et al., 2022; Yang et al., 2023). GAS, the primary bioactive constituent of Gastrodia elata (Liu et al., 2022), demonstrates renoprotective effects against ischemia-reperfusion injury by suppressing tubular apoptosis (Zheng et al., 2022). In cardiovascular diseases, GAS exhibits not only the beneficial effects of lowering blood pressure, regulating blood lipids, and anti-thrombosis (Ahmad et al., 2019), but it has also been demonstrated to diminish myocardial hypertrophy and cardiac fibrosis (Shu et al., 2012). GAS further mitigates MIRI by modulating expression of apoptotic regulators and inflammatory cytokines (Han et al., 2019). Specifically, recent studies have revealed that GAS exerts a notable protective effect in MIRI by regulating inflammatory factors (Zhang H.-S. et al., 2019), anti-oxidative stress (Song et al., 2016), regulating autophagy (Fu et al., 2018; Rao et al., 2022), mediating cell death (Liu et al., 2016). Despite extensive mechanistic validation, GAS’s precise molecular targets in MIRI remain incompletely characterized.

CNPase (2′,3′-cyclic nucleotide-3′-phosphodiesterase) localizes between mitochondrial membranes (Azarashvili et al., 2009; 2014), catalyzing 2′,3′-cyclic nucleotides as substrates (Heaton and Eckstein, 1996), which has been identified to impair mitochondrial integrity and accelerate the opening of the mitochondrial permeability transition pore (mPTP) (Azarashvili et al., 2009). The opening of mPTP can activate apoptotic pathways (Bonora et al., 2022). Elevated CNPase expression during heart failure augments cardiac protection (Baburina et al., 1859; Odinokova et al., 2018), though its role in MIRI remains undefined. As early as 1989, researchers’ immunofluorescence results reveal CNPase distribution extends beyond membrane-proximal regions to include tubulin binding within oligodendroglial networks and direct cytoskeletal associations with F-actin and tubulin (Dyer and Benjamins, 1989). It was also found that CNPase anchors myelin-related proteins to the cytoskeleton (Barradas and Cavalcante, 1998). CNPase interacts with the cytoskeleton of actin to counteract the developmental closure of cytoplasmic channels transmitted through dense myelin (Markusson et al., 2024).

TG2, an endothelial cell cytoskeletal regulatory factor (Xiao et al., 2012) and a member of the calponin protein family, demonstrates high expression in smooth muscle cells (Rao et al., 2015). Its biochemical and biological properties position TG2 as a promising pharmacological target across multiple diseases contexts (Meng et al., 2017; Yin et al., 2018). Study revealed that TG2 serves as a promising therapeutic target for addressing pulmonary resistance in asthma (Yin et al., 2018). Besides, the expression and phosphorylation of TG2 correlate with poor cancer prognosis (Yin et al., 2019). Transgelin protein may be an indicator of the development of pulmonary arterial hypertension related to congenital heart disease and a potential therapeutic target for the treatment of pulmonary arterial hypertension related to congenital heart disease (Zhou et al., 2022). Mechanistically, membrane-localized TG2 enhances actin stability (Goodman et al., 2003; Na et al., 2015). Nevertheless, the relationship between TG2 and CNPase remains unclear. It is worth noting that the effect of TG2 on MIRI awaits elucidation.

Therefore, our study has demonstrated that GAS confers protection against MIRI through modulation of the TG2/CNPase pathway. In this study, we establish that GAS treatment attenuates MIRI-mediated myocardial injury and apoptosis. Mechanistically, CNPase expression was found to increase during MIRI, which was reversed by GAS therapy. Additionally, CNPase was identified to combine with TG2, whose expression decreased in response to MIRI. TG2 negatively regulated the expression of CNPase. Crucially, CNPase deficiency reduces apoptosis and ameliorates MIRI pathology. These findings provide both mechanistic insight into GAS-mediated cardioprotection and identify CNPase as a novel therapeutic target for MIRI intervention. The main workflow of this study is presented in Supplementary Figure S3.

## 2 Materials and methods

### 2.1 Cell experiments

The H9c2 cell line (Bei na Chuang lian Biology) was cultured with DMEM/F12 culture medium (GIBCO) containing 10% fetal bovine serum (GIBCO) and 1% double antibodies (penicillin and streptomycin) (Hyclone) at 37°C in a humidified 5% CO_2_ atmosphere. Cells at 70%–80% confluency were washed thrice with PBS and subjected to oxygen-glucose deprivation (OGD) by incubation in serum-free, low-glucose medium (GIBCO) within a 1% O_2_/5% CO_2_/94% N_2_ chamber at 37°C for 2 h, followed by normoxic recovery. For pretreatment, the GAS pretreatment group underwent treatment with 10 μM (μM) GAS for a duration of 30 min (min) prior to the deprivation of oxygen and glucose. We established a concentration gradient of GAS and identified 10 μM as the optimal dosage through assessment of cell morphology and viability.

### 2.2 Animal experiments

Male Sprague Dawley (SD rats) (250 ± 20 g) were provided by Kunming Medical University [Animal Permit No. SCXK (DIAN) K2020-0004]. All animal care procedures and laboratory procedures are conducted according to the guidelines of the Animal Ethics and Laboratory Committee of Kunming Medical University and following the Guidelines for the Care and Use of Laboratory Animals (revised in 1996). The animals were kept at controlled temperatures (22°C ± 1°C) and humidity (45%–55%) on a 12 h light-dark cycle and in an environment free of specific pathogens. Give standard rodents food and water. Rats were anesthetized by intraperitoneal injection of 1% pentobarbital sodium (30 mg/kg) (Wang et al., 2022). Ischemia/reperfusion (I/R) operation was performed on SD rats. The SD rats were anesthetized by intraperitoneal injection of pentobarbital sodium, and connected to the small animal ventilator after oral intubation. Electrocardiogram changes were monitored by the double-lead ECG monitor. The hair on the rats’ chests was removed, followed by rigorous disinfection. The skin was then incised along the fourth rib on the left margin of the sternum to expose the chest cavity. The heart was fully revealed, and the pericardium was subsequently torn open. The left anterior descending coronary artery was exposed and lapped. The heart tissue below the ligation site turns white. ST-segment elevation in II lead ECG indicated the success of the model. 45 min after ligation (Ischemia duration was standardized to 45 min based on clinical translation relevance (Agewall et al., 2017) and previous literature support (Rao et al., 2022) to ensure reproducible infarction with controlled apoptosis), the lapped anterior descending branch of the left coronary artery was released at different time points (0 h, 20 min, 2 h, 8 h, 12 h, 24 h) (Rao et al., 2022) for reperfusion. In the sham operation group, only the heart was exposed, the pericardium was torn, and the left anterior descending coronary artery was not ligation. In the ischemic group, no reperfusion was carried out subsequent to the ligation of the left anterior descending coronary artery for a duration of 45 min. The GAS treatment group was treated 3 days in advance. GAS was intraperitoneally injected into SD rats for three consecutive days at concentrations of 100 mg/kg/day. The dosage of Gastrodin *in vivo* was determined with reference to clinical trial protocols for Gastrodin (Registration No.: ChiCTR1800020414), applying the established human-to-rat dose conversion formula: X mg/kg × 70 kg × 0.018/200 g = 6.3 X mg/kg. This conversion methodology, based on body surface area normalization between species, is a well-validated approach (Xiao et al., 2023; Chen, 2024). To ensure the rigor of the experiment, we used 100 mg/kg/day as the sole dose in the full-text *in vivo*. I/R operation was performed on SD rats after GAS treatment.

### 2.3 Langendorff isolated cardiac perfusion system

SD rats who completed the MIRI model were exposed the aortic arch and the heart was clipped along the arch after anesthesia. Immediately place the heart in a pre-cooled K-H solution (0°C–4°C) and gently press the heart with your hand to remove residual blood. The gas in the Langendorff tube was pre-drained, the perfusion tube was inserted into the aorta, the heart was fixed, and then retrograde perfusion was officially started. Create a minute incision adjacent to the right atrium to facilitate the drainage of perfusion. Once the Langendorff isolated heart perfusion model is established, a steady state is maintained, featuring a constant temperature of 37.5°C, a constant pressure ranging from 60 to 70 mmHg, and a continuous supply of oxygenated perfusion fluid with a mixture of 95% O2 and 5% CO2. Heartbeat again, good heartbeat, after meeting the inclusion criteria to start the group experiment. Inclusion criteria: arrhythmia, heart rate (HR)≥200 beats/min, left ventricular developing pressure (LVDP)≥60 mmHg. Cardiac function was assessed by monitoring: HR, left ventricular systolic pressure (LVSP), LVDP, rate-pressure product (RPP), and stroke index (SI).

### 2.4 TTC staining

After the successful construction of the I/R model, PBS performed cardiac perfusion and collected the hearts. Place the hearts in an ultra-low temperature refrigerator at −80°C for 10 min, and cut the hearts into 2–3 mm thick slices with a blade. The 2% 2,3,5-triphenyltetrazolium chloride TTC solution was used to immerse the hearts slices at 37°C for 30 min. Turn the heart sections properly to contact fully with the staining solution. The white part shows the infarct area. Subsequently, the heart sections were fixed with 4% paraformaldehyde for 10 min, photographed with a digital camera, and the infarct size was analyzed with ImageJ software.

### 2.5 H&E staining

Following successful induction of myocardial ischemia-reperfusion in SD rats, hearts were excised and immersion-fixed in 4% paraformaldehyde (PBS-buffered) for 24 h at 4°C. Fixed tissues were dehydrated through an ethanol series, cleared in xylene and embedded in paraffin in blocks. Serial sections (5 μm thickness) were cut using a rotary microtome (Leica RM2235) mounted on charged slides, and dried overnight at 37°C. After deparaffinization in xylene and rehydration through graded alcohols, sections were stained with hematoxylin (Solarbio) for 8 min and eosin Y (Solarbio) for 1 min according to manufacturer’s protocol. Stained sections were dehydrated, cleared, and permanently mounted with neutral balsam. Take a picture.

### 2.6 Immunofluorescence analysis

Following deparaffinization in xylene (3 × 5 min) and rehydration through graded ethanol series (100%–70%), endogenous peroxidase activity was quenched with 3% H_2_O_2_ for 15 min at room temperature. After three 5-min distilled water washes, antigen retrieval was conducted in EDTA buffer (PH 8.0) (Servicebio) using a microwave decloaking chamber (95°C, 20 min). Sections were cooled to room temperature (30 min) and rinsed in distilled water (3 min). The tissue was added with the 5% bovine serum albumin (BSA) (Servicebio) solution and sealed for 30 min. About 20 μL anti-TG2 (Cell Signaling Technology, 1:200) or anti-PCBP1 (Proteintech, 1:200) or anti-CNPase (Proteintech, 1:200) antibody was added to the tissue and kept at 4°C overnight. The next day, wash the sections with PBS buffer containing Tween-20 (PBST) 5 times for 5 min each time. The prepared fluorescent secondary antibody (anti-rat-488 or anti-rabbit-594) (Proteintech, 1:500) was added to the tissue and incubated at room temperature or 37°C for 2 h, away from light. Then wash the sections with PBST 5 times for 5 min each time. In a dark room, about 10 uL anti-fluorescence attenuation sealer (containing DAPI) (Beyotime Biotechnology) is added to the tissue on the slide, then the cover glass is covered to avoid bubbles. The slices were placed in an anhydrous capsule, and once they were thoroughly dried, the resulting images were examined under a fluorescence microscope.

### 2.7 Cells immunofluorescence assay

Once the H9c2 cell model was established, the cells underwent three thorough rinses with PBS buffer. They were then covered with 4% paraformaldehyde and fixed for a duration of 15 min. Following this, the cells were again rinsed three times with PBS, with the fixing solution subsequently removed. The cells were then incubated with 0.3% Triton X-100 (Biofroxx) for 10 min, rinsed three more times with PBS, and finally blocked with 5% BSA at 37°C for 30 min. The blocking solution was removed and an anti-CNPase antibody (1:200) was added for overnight incubation at 4°C. The next day, clean with PBST buffer 3 times for 5 min each time, add fluorescent secondary antibody and F-actin to co-incubate for 2 h, and clean with PBST buffer 3 times for 5 min each time. The cell slipper, positioned at the base of the orifice plate, was removed and securely fastened in reverse onto the slide that contained the DAPI anti-fluorescence attenuation tablet. The assembly was then placed in a dark environment, and once the tablet had air-dried, photographs were taken.

### 2.8 Rhodamine 123 staining

When the H9c2 cells fusion degree reached 60%–70%, Rhodamine 123 solution (Merck) was added to the cell culture medium to make the final concentration of 10 μM. H9c2 cells were incubated with Rhodamine 123 for 30 min. Remove the staining solution and the cells were cleaned with PBS buffer. The cells were incubated with the hochest 33,342 staining solution (Beyotime Reagent Company) for 10–20 min. Remove hochest 33,342 staining solution and clean cells with PBS. Images were taken using an inverted fluorescence microscope.

### 2.9 Tunel assay

H9c2 cardiomyocytes were cultured on the climbing tablets. After the H9c2 cell density reached 60%–70%, the cells were cleaned three times with PBS, then fixed with 4% paraformaldehyde, and the fixing fluid was washed away with PBS. The cells were then incubated with 0.3% triton X-100 for 5 min and cleaned with PBS. About 50 μL Tunel staining solution (Beyotime Reagent Company) was added to the cell sample and incubated for 0.5–1 h away from light. The cell slides were then removed and sealed with anti-fluorescence attenuation tablets containing DAPI (Beyotime Reagent Company). Take pictures.

### 2.10 Flow cytometry

When H9c2 cells were fused to 60%–70%, the cells were cleaned with PBS 3 times, digested, and centrifuged to remove the supernatant and collect the cells. 50,000 to 100,000 cells were taken and centrifuged at 1000 *g* for 5 min. The supernatant was discarded, and the cells were gently resuspended with 195 μL Annexin V-FITC binding solution. Add 5 μL Annexin V-FITC and mix gently. Add 10 μL propyl iodide staining solution and mix gently. Incubate at room temperature (20°C–25°C) away from light for 10–20 min, then place in an ice bath. Annexin V-FITC kit was purchased from Beyotime Reagent Company. Aluminum foil can be used for light protection. Flow cytometry was used for detection.

### 2.11 Gene silencing and overexpression experiment

The overexpression plasmid of TG2, PCBP1, and the siRNA of TG2, PCBP1, and CNPase were purchased from GenePharma Co., Ltd. The cells were implanted into a 6-well plate. Transfection was performed when the cell fusion reached 60%–70%. After thoroughly washing the cells with PBS buffer three times, a serum-free, antibiotic-free base medium was added to each well, followed by incubation for 8–12 h. Subsequently, 5 μL plasmid and 5 μL Lipofectamine™ 2000 (Invitrogen) transfection reagent was mixed in 250 μL of serum-free medium, incubated for 20 min, and then gently introduced into a 6-well plate. After 12 h, the transfection compound was replaced with a normal growth medium. Then, analyzed the protein expression.

### 2.12 Cell morphology and viability

Cell viability assessments included different concentration GAS (0–1000 μM)-treated groups. In this setup, the 0 μM group served as the control, with H9c2 cells treated using complete medium containing only the Gastrodin vehicle solvent dimethyl sulfoxide (DMSO) without GAS itself. Cell morphology was observed under microscope. When the growth and fusion rate of H9c2 cells reached 80%–90%, they were digested with trypsin and then transferred to 96-well culture plates for culture. The next day, 10 μL of CCK-8 reagent were added to each well. After incubation for 1 h, the cell viability was measured by colorimetry. Another 96-well cell culture plates were taken, centrifuged at 400 *g* for 5 min, and 60 μL LDH working solution was added to each well for analysis, and incubated at 37°C in the dark for 30 min. The optical density of CCK8 is 450 nm and that of LDH is 490 nm, which were measured using an enzymoleter.

### 2.13 Protein-protein molecular docking

Download the crystal structures of TG2 and CNPase from the PDB database (https://www.rcsb.org/). Upload the crystal structure of TG2 and CNPase to the molecular docking site (https://gramm.compbio.ku.edu/gramm). The obtained docking results were opened using the Pymol software. The molecular docking was carried out to demonstrate the docking hydrogen bond and measure the hydrogen bond distance.

### 2.14 Immunocoprecipitation (Co-IP) technology

In this study, the Co-IP experiment was used to detect the interactions between proteins. H9c2 cells were cultured in a 10 cm^2^ dish. The lysate obtained from the co-immunoprecipitation kit was introduced into the cell culture dish. It was then subjected to cleavage on ice for a duration of 30 min, utilizing a sterile cell scraper. Following this, the lysate was transferred into a clean 1.5 mL centrifuge tube and subjected to further cleavage on ice for another 30 min, with gentle inversion mixing every 10 min to ensure thorough mixing. After lysis, the centrifuge tube containing cell suspension was centrifuged (12,000 rpm, 30 min), and the cell supernatant was divided into 1/6 input, IgG group, and target protein group, which were transferred to the new centrifuge tube. TG2 antibody (rabbit origin, proteintech) or CNPase antibody (mouse origin, proteintech) was added to the target proteome, and the same amount of control IgG antibody (proteintech) of the same origin as the target proteome was added to the IgG group. Then protein A and G were added and incubated at 4°C for 12 h. The precipitate was washed with the washing solution in the co-immunoprecipitation kit, and 20–40 µL 1* sampling buffer was added. This was followed by Western blot analysis.

### 2.15 Western blot (WB) analysis

The total protein of heart tissue and H9c2 cells was extracted with high-efficiency RIPA protein lysate. The BCA protein content assay kit (Beyotime Institute of Biotechnology) was used to determine the protein concentration. SDS-PAGE glue was used to separate the equal concentration of proteins, then the proteins on the glue were transferred to the PVDF membrane (Millipore), and the membrane was sealed with 5% skim milk prepared by Tris-Buffered Saline with Tween-20 (TBST) for 2 h. The membrane was then incubated with anti-TG2, PCBP1 CNPase, Cleaved-Caspase 3, Bax, BCL2, and β-Actin specific antibodies at 4°C overnight. The next day, TBST buffer was used to clean the membrane 5 times for 5 min each time. A secondary antibody of the same genus as the specific primary antibody was then used to incubate the membrane at room temperature for 2 h. Continue to wash with TBST 5 times, 5 min each time. The chemiluminescence solution was added, the chemiluminescence imager monitored the protein expression, and the ImageJ software quantified the protein.

### 2.16 Statistical analysis

All statistical analyses were performed using GraphPad Prism 8.0.1 software (Inc., La Jolla, San Diego, CA, United States). The data are expressed as the mean standard deviation (SD) from at least three replicates. Sample sizes were based on empirically established standards for rodent MIRI models (Lee et al., 2012; Shen et al., 2024). Based on pre-experimental data, the effect size (Cohen’s d) was calculated as 21.47. Youdaoplaceholder0 an extremely large effect. Post-hoc power analysis using G*Power showed >99.9% power to detect this difference at α = 0.05 confirming the feasibility of n = 3. All data points represent biological replicates from independent experiments. Paired T-tests for single-factor comparisons with two groups should be used. Univariate and multi-group comparisons were made using univariate ANOVA and Tukey *post hoc* test. All ANOVA analyses proceeded only after confirming non-significant Levene’s results (p > 0.05). Then the Tukey test is performed. The error bar represents the SEM for all experiments. When *P < 0.05, the value was considered statistically significant. ns, not significant.

## 3 Result

### 3.1 GAS improves cardiac dysfunction induced by MIRI

**FIGURE 1:**
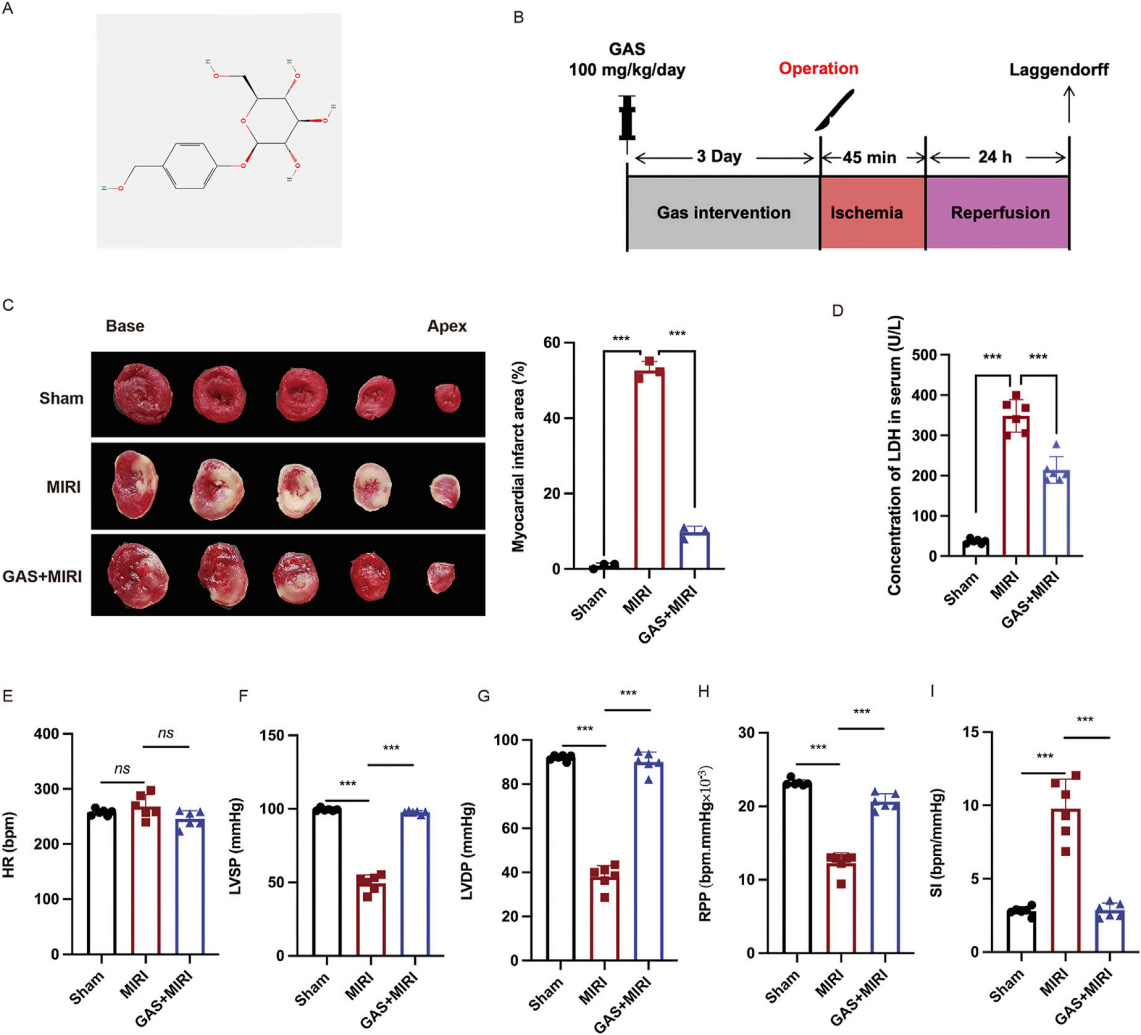
GAS reduces ischemic area and improves cardiac dysfunction. **(A)** Molecular structure of GAS. **(B)** Animal experiments procedure. **(C)** TTC staining was used to detect myocardial infarction area. n = 3. **(D)** Serum LDH levels in SD rats. n = 6. **(E-I)**. The Langendorff isolated cardiac perfusion system was applied to exam the cardiac function including HR **(E)** LVSP **(F)**, LVDP **(G)**, RPP **(H)**, SI **(I)**. n = 6. *p < 0.05, **p < 0.01, ***p < 0.001, ns, not significant.

GAS is a traditional Chinese herbal medicine whose protective effect against MIRI has been proven (Chen, 2024). The chemical structure formula of GAS is shown in Figure 1A (Figure 1A). To investigate the effect of GAS on MIRI, we administered GAS intraperitoneally to SD rats for three consecutive days. Subsequently, a classical model of ligation and recanalization of the left anterior descending coronary artery (Lv et al., 2022) was induced (Figure 1B). TTC staining was applied to assess the size of the myocardial infarction. Compared to the sham group, the myocardial infarction territory in the I/R group demonstrated a statistically significant enlargement (Figure 1C). However, the pretreatment with GAS resulted in a reduction of the myocardial infarct area caused by I/R (Figure 1C). LDH is a biomarker of MIRI in serum. We examined serum LDH levels in SD rats and the results showed that LDH concentrations were elevated in the MIRI group compared to the sham group. However, GAS preconditioning reduced the high level of MIRI mediated LDH with statistical significance (Figure 1D), indicating that GAS can reduce myocardial injury induced by MIRI. We further measured the cardiac function of SD rats using the Langendorff isolated cardiac perfusion system. The cardiac systolic function, including LVSP (Figure 1F), LVDP (Figure 1G), RPP (Figure 1H) were significantly decreased in the I/R (I-45 min, R-24 h) group, which was restored by the pretreatment with GAS. The pretreatment of GAS also improved the decrease of SI induced by I/R (Figure 1I). However, the changes of HR in the three groups have no statistical difference (Figure 1E). These results indicated that the pretreatment of GAS alleviated the cardiac function damage caused by I/R.

### 3.2 GAS treatment alleviates myocardial cell damage

**FIGURE 2:**
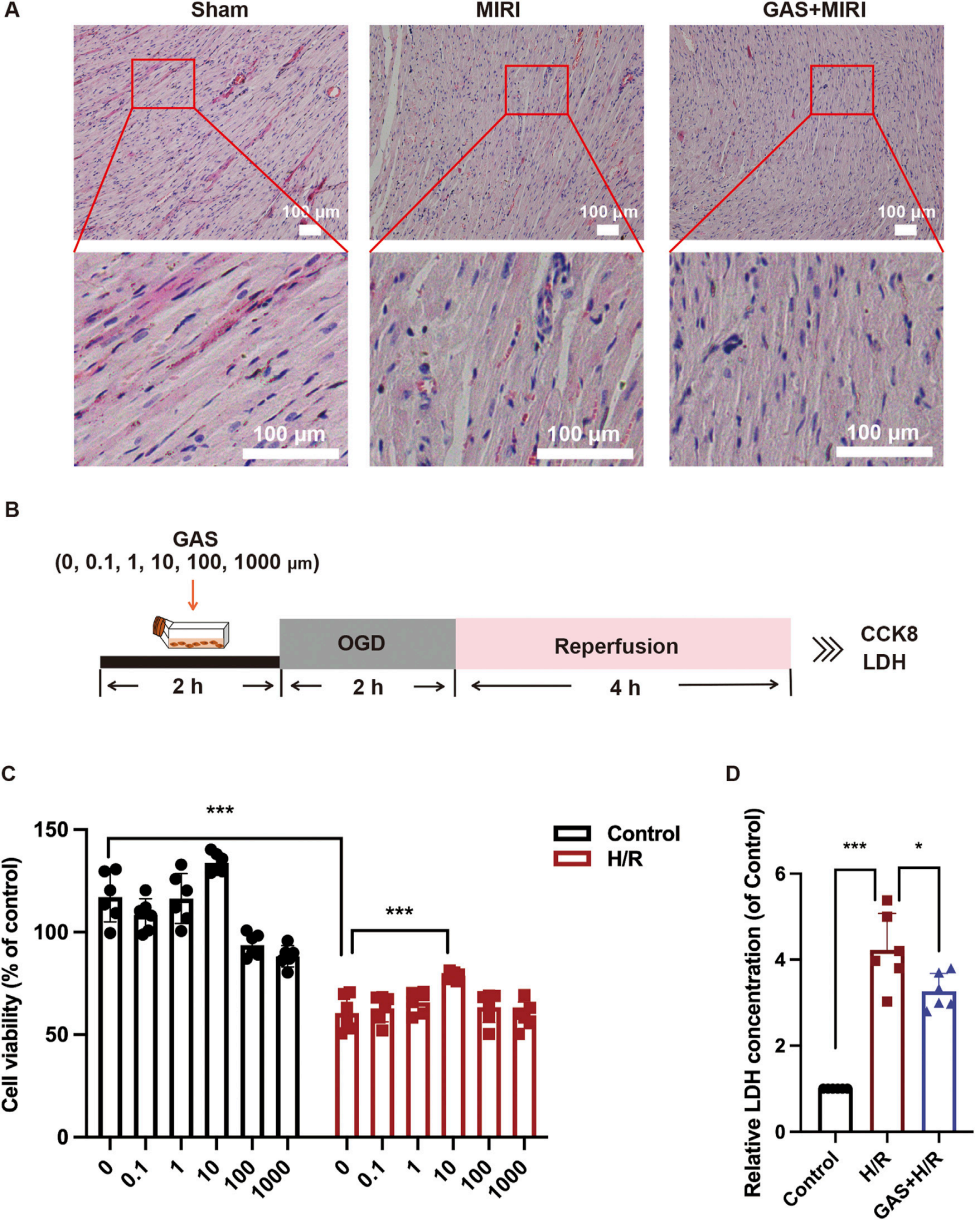
GAS alleviates MIRI mediated cardiomyocyte damage. **(A)** Myocardial injury was detected by HE staining. Scale bar = 100 μm. n = 3. **(B)** The OGD model of H9c2 cells was constructed. **(C)** CCK-8 was used to detect the cytotoxicity of GAS. n = 6. **(D)** LDH levels in H9c2 cell medium. n = 6. *p < 0.05, **p < 0.01, ***p < 0.001, ns, not significant.

Subsequently, we examined the effect of GAS on the myocardial cell damage caused by MIRI. Cross-sectional sections of the hearts of each group of SD rats were examined for changes in cell and tissue structure using H&E staining. Compared with the sham group, the myocardium in SD rats with MIRI showed pathological myocardial fiber disturbance in the infarction area, which was characterized by typical necrotic transformation with myoplasmic consolidation and disappearance of transverse stripes. The pathological injury of myocardium in GAS pretreated group compared with the MIRI group was significantly improved, the arrangement of cardiomyocytes was orderly, the apoptosis of cardiomyocytes was reduced, and the infiltration of neutrophils in myocardium was significantly reduced (Figure 2A). To examine the effect of GAS on cardiomyocytes, we constructed a classical *in vitro* H/R model using stable rat cell line H9c2 cells (Figure 2B). We evaluated the cytotoxicity of GAS by CCK8 cell viability assay and investigated whether it protects H9c2 cells from MIRI-induced damage. The results showed that H/R injury resulted in a significant decrease in H9c2 cell activity, while GAS pretreatment partially restored cell vitality, of which 10 μM GAS pre-administration was the best dose (Figure 2C). Therefore, we selected 10 μM as the dose of GAS to pretreat H9c2 cells in subsequent *in vitro* experiments. The H/R model was constructed after GAS pre-administration on H9c2 cells, and the concentration of LDH secreted in the supernatant of cell culture was detected to identify the damage of H9c2 cells. It was found that GAS pretreatment reversed the H/R-mediated increase in LDH levels (Figure 2D). These results collectively demonstrated that GAS pretreatment repaired cardiomyocytes hypoxic reoxygenation injury induced by MIRI.

### 3.3 GAS preconditioning improves MIRI-mediated apoptosis

**FIGURE 3:**
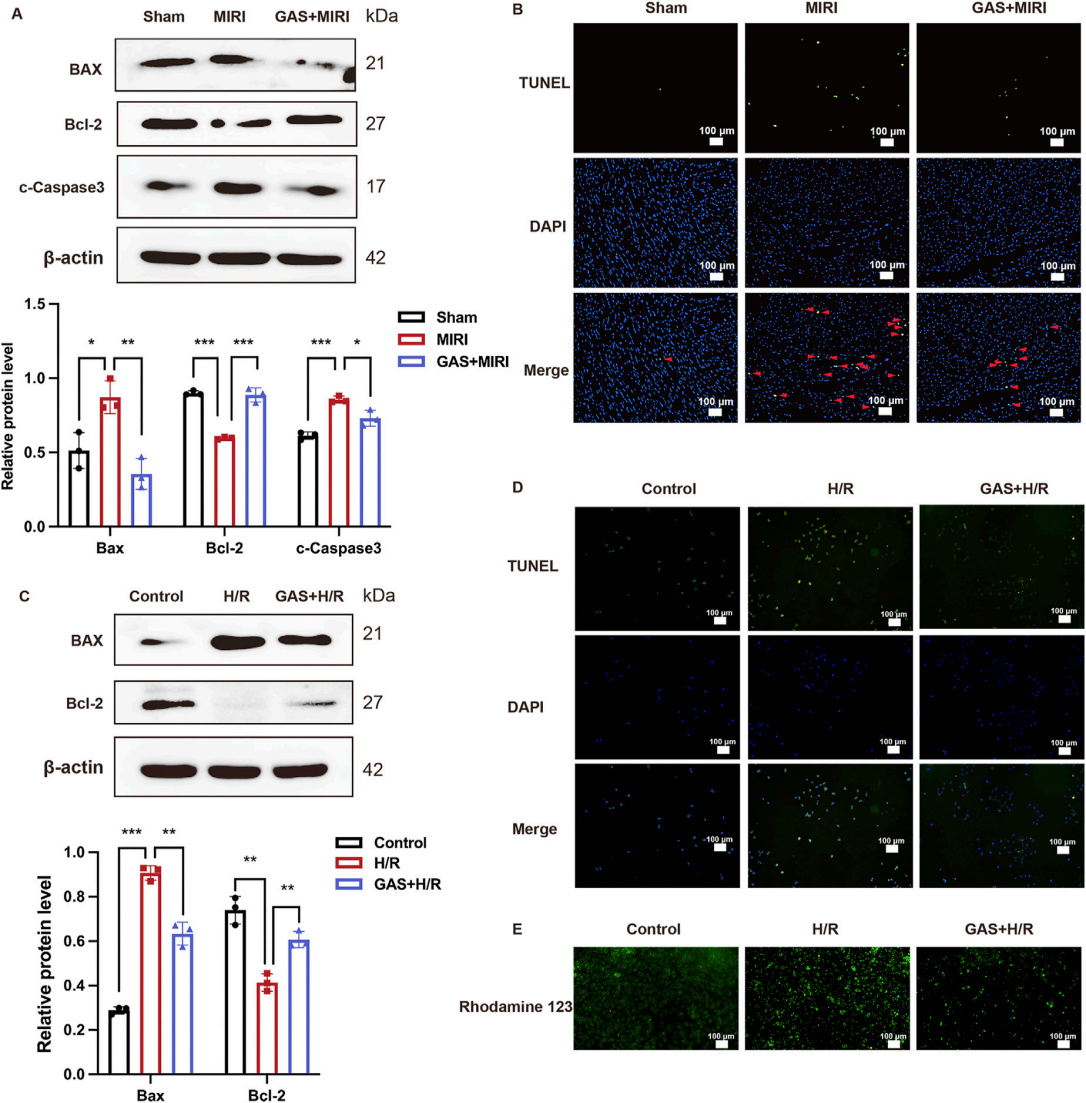
GAS therapy improved apoptosis induced by MIRI. **(A)** WB detect the expression of apoptosis-related proteins *in vivo*. n = 3. **(B)** TUNEL staining of myocardial tissue. n = 3. **(C)** WB analysis and quantification of apoptosis-related proteins *in vitro*. n = 3. **(D)** TUNEL staining of H9c2 cells. n = 3. **(E)** Rhodamine 123 staining *in vitro*. n = 3. *p < 0.05, **p < 0.01, ***p < 0.001, ns, not significant.

In previous results, we found that GAS pretreatment improved cardiac dysfunction and cardiomyocyte damage caused by MIRI. Next, we conducted an in-depth exploration of the potential mechanism by which GAS protects MIRI. *In vivo*, we pretreated SD rats with GAS and constructed MIRI models to detect the expression of apoptosis-related proteins. We detected significantly increased protein expression of Bax and c-Caspase3 in myocardial tissue of SD rats with MIRI, while decreased expression of Bcl-2, suggesting that MIRI leads to increased apoptosis in myocardial tissue. Compared with the MIRI group, the expression of apoptosis-related proteins (Bax and c-Caspase3) decreased in the GAS pretreatment group, while the expression of Bcl-2 was relatively enhanced (Figure 3A). At the same time, the myocardial tissues of SD rats in different groups were sliced. The apoptosis was detected using TUNEL detection kit. The results demonstrated that compared with the sham group, more apoptosis was detected in the myocardial tissue of MIRI group, while the increase in apoptosis caused by MIRI was reduced by GAS preconditioning (Figure 3B; Supplementary Figure S1A).

*In vitro*, H9c2 cells were deprived of oxygen and glucose in order to construct the H/R models, and GAS was added in advance for pretreatment, and then the apoptosis level of H9c2 cells in different groups was detected. First, the detection results of apoptosis-related proteins showed that H9c2 cells in the H/R group expressed more Bax and c-Caspase3, while the expression level of Bcl-2 protein was relatively reduced compared with the control group. However, GAS pretreatment mitigated the differences in apoptosis-related protein expression caused by H/R. The protein expression of Bax and c-Caspase3 in GAS treatment group was lower than that in H/R group, while the protein level of Bcl-2 was higher (Figure 3C). The TUNEL staining results of H9c2 cells were consistent with the WB results, indicating that GAS pretreatment mitigated the increase of apoptosis caused by H/R (Figure 3D; Supplementary Figure S1B). This result was also confirmed by Rodamine 123 staining. Compared with the control group, H9c2 cells showed more apoptosis after H/R treatment, while GAS pretreatment alleviated the apoptosis of H9c2 cells (Figure 3E; Supplementary Figure S1C). These results suggest that GAS preconditioning can effectively improve the increase of apoptosis mediated by MIRI.

### 3.4 GAS alleviates the CNPase increased caused by MIRI

**FIGURE 4:**
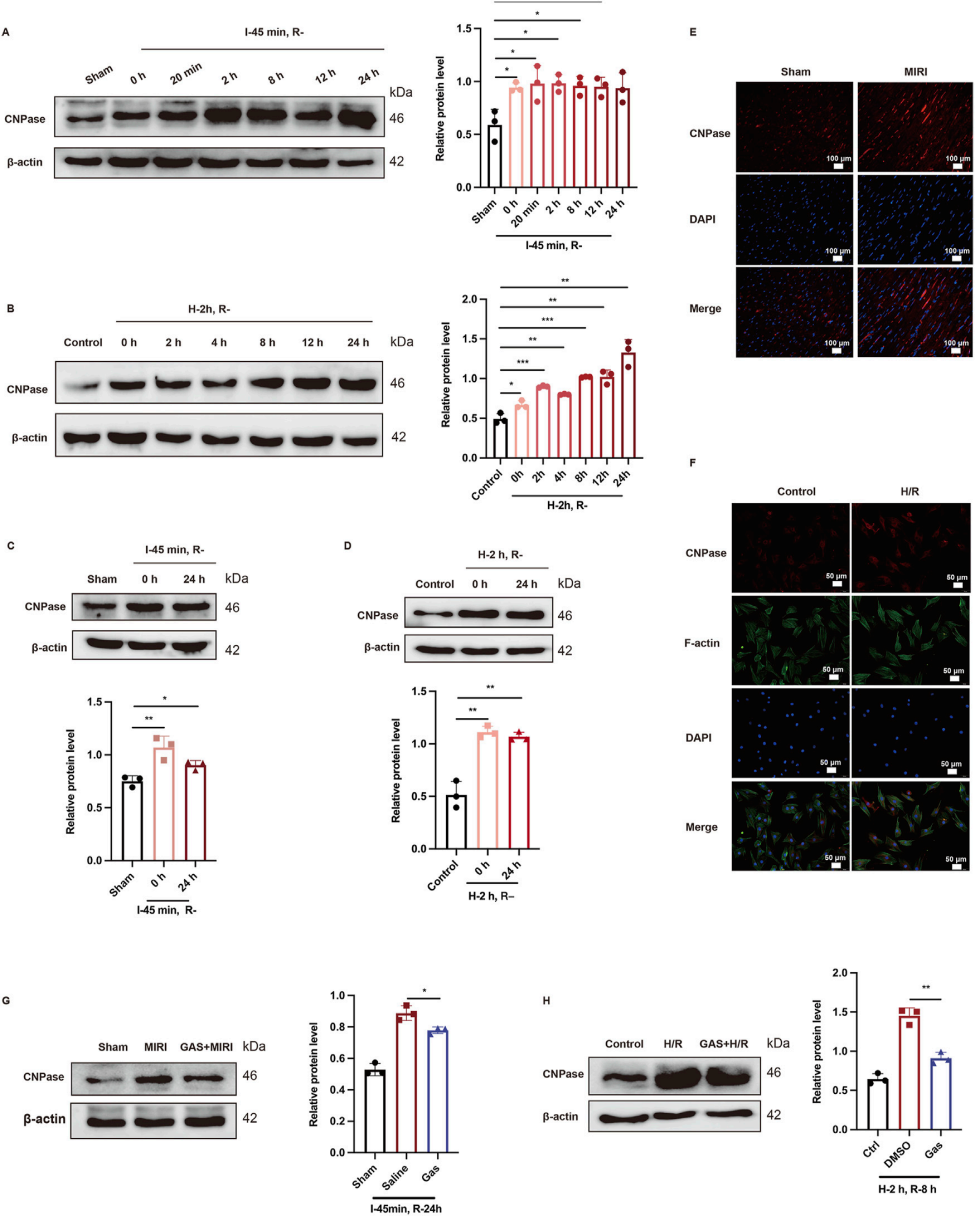
GAS preadministration reversed MIRI induced CNPase increase. **(A,B)** Representative WB analysis and quantification of CNPase at different reperfusion time points *in vitro* **(A)** and *in vivo* **(B)** n = 3. **(C,D)** WB analysis was used to detect the protein level of CNPase in rats when I/R (45 min/24 h) **(C)** or in H9c2 cells when H/R (2 h/24 h) **(D)** Data are presented as relative fold change to Control or Sham. n = 3. **(E)** IF detected the CNPase in myocardial tissue of rats. n = 3. Scale bar = 100 μm. **(F)** IF detected the CNPase *in vitro*. n = 3. Scale bar = 100 μm. **(G,H)** WB analysis detected the effect of GAS pretreatment on the expression of CNPase *in vivo* **(G)** and *in vitro* **(H)** Results are means ± SEM of three independent experiments. n = 3. *p < 0.05, **p < 0.01, ***p < 0.001, ns, not significant.

In recent years, CNPase has been identified as a novel heart failure treatment strategy (Wang et al., 2021) and has been discovered to alleviate heart hypertrophy by enhancing mitochondrial energy production (Tan et al., 2021). Nevertheless, the role of CNPase in MIRI remains undiscovered. Here, we administered SD rats with ischemia for 45 min, followed by reperfusion for 0 h, 20 min, 2 h, 8 h, 12 h, and 24 h. Then the protein expression level of CNPase was detected using WB. The protein level of CNPase rose with the prolongation of reperfusion time (Figure 4A). Specifically, the protein expression of CNPase was the highest at 24 h after reperfusion (Figure 4A). *In vitro*, H9c2 cells were cultured for 2 h with low glucose medium and a hypoxia condition, and then returned to normal culture conditions for 0 h, 2 h, 4 h, 8 h, 12 h, and 24 h, respectively. Consistent with the results *in vivo*, compared with the control group, the expression of CNPase increased with the extension of reperfusion time, and the expression of CNPase was the highest at 24 h of reperfusion culture (Figure 4B). Subsequently, GAS preconditioning was performed on SD rats, and MIRI models with ischemia for 45 min and reperfusion for 24 h were constructed to detect the protein expression level of CNPase in myocardial tissue. CNPase protein expression was observed to increase markedly in MIRI group (Figure 4C). Immunofluorescence staining of CNPase in myocardium of different groups also suggested that the expression of CNPase in myocardium of MIRI group was higher than that of sham group (Figure 4E; Supplementary Figure S1D). For H9c2 cells, we also established H/R model by oxygen-glucose deprivation for 2 h, then reperfusion for 24 h. Then we found that CNPase expression was obsevably increased in the H/R group compared with the control group (Figure 4D). Similarly, IF staining of H9c2 cardiomyocytes (Figure 4F; Supplementary Figure S1E) indicated that the protein fluorescence intensity of CNPase during H/R was higher than that of the control group, indicating that the expression of CNPase increased during ischemia-reperfusion injury, which is consistent with the results *in vivo*.

Subsequently, the effect of GAS on CNPase was explored. GAS was introduced to H9c2 cells for a duration of 30 min prior to the induction of H/R. The impact of GAS pretreatment on the expression levels of CNPase was then assessed. Our findings revealed a decrease in CNPase expression upon the addition of GAS (Figure 4G). Conformably, compared with the MIRI group, GAS preconditioning reduced the I/R-mediated increase of CNPase (Figure 4H). The findings suggested that there is an elevation in the expression of CNPase in MIRI, whereas GAS preconditioning has the ability to counteract the MIRI-induced upregulation of CNPase.

### 3.5 GAS reversed the reduction of TG2 levels induced by MIRI

**FIGURE 5:**
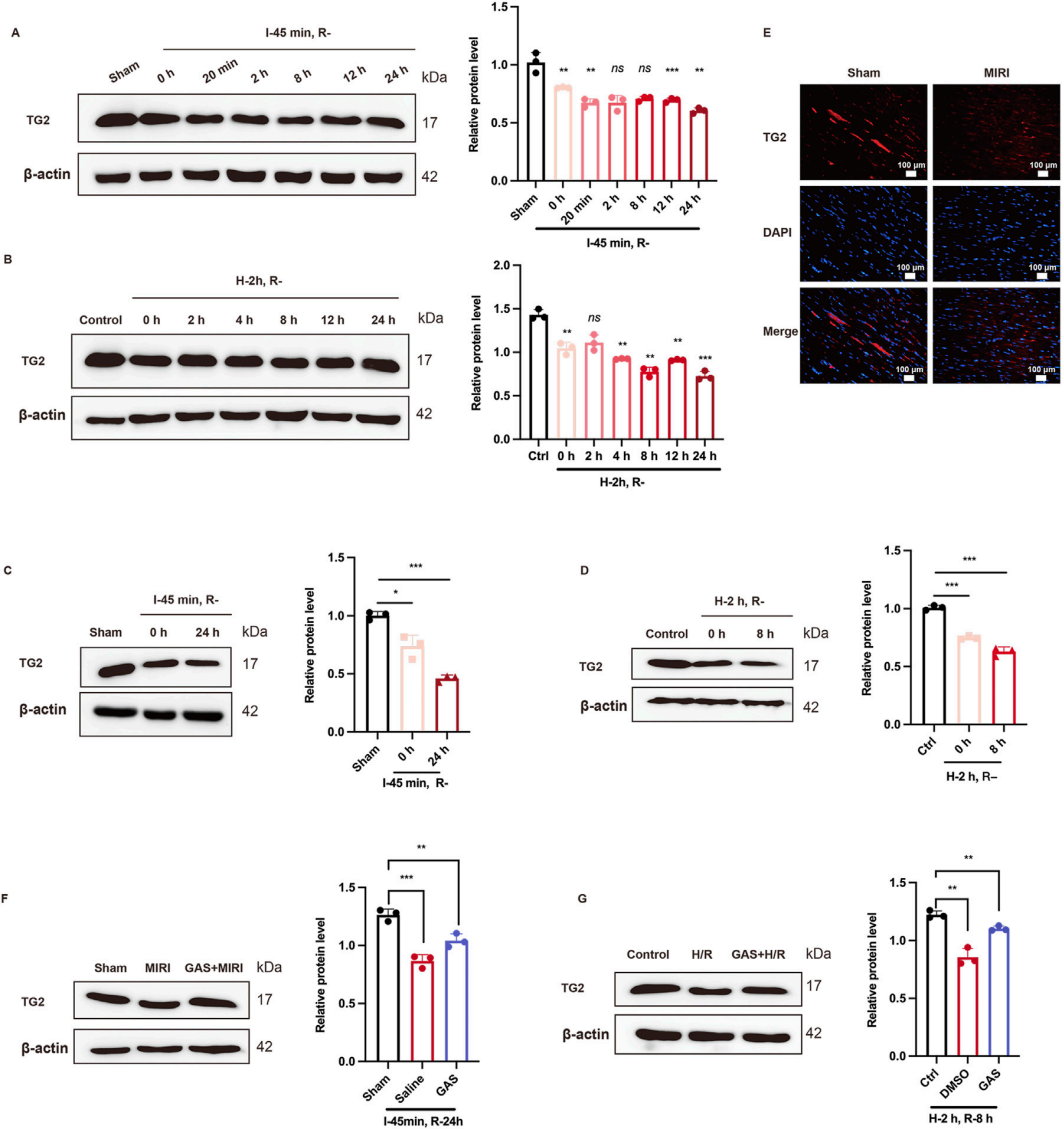
GAS changes TG2 reduction due to MIRI. **(A,B)** WB detect the expression of TG2 at different reperfusion time points *in vitro* **(A)** and *in vivo* **(B)** n = 3. **(C,D)** WB was used to detect the protein level of TG2 *in vivo* **(C)** and *in vitro* **(D)**. Data are presented as relative fold change to Control or Sham. n = 3. **(E)** IF detected TG2 in myocardial tissue of rats. n = 3. Scale bar = 100 μm. **(F)** and **(G)**. WB analysis detected the protein level of TG2 after GAS pretreatment *in vivo* **(F)** and *in vitro* **(G)**. Results are means ± SEM of three independent experiments. n = 3. *p < 0.05, **p < 0.01, ***p < 0.001, ns, not significant.

TG2, a member of the transgelin family, has been confirmed to be an actin-binding protein induce actin gelation and regulate the actin cytoskeleton (Yin et al., 2019). However, the role of TG2 in MIRI has not been reported. Exploring the effect of TG2 on MIRI may provide a robust theoretical basis for the prevention, diagnosis, and treatment of MIRI. In this study, we used SD rats to build MIRI model by ischemia for 45 min, followed by reperfusion for 0 h, 20 min, 2 h, 8 h, 12 h, and 24 h. The protein expression level of TG2 as the reperfusion time was declined (Figure 5A). Specifically, the expression level of TG2 was at its lowest point at 24 h of reperfusion (Figure 5A). Furthermore, WB was used to detect the TG2 expression of myocardial tissue in SD rats after 45 min ischemia and 24 h reperfusion, which results found that TG2 decreased evidently when MIRI occurs (Figure 5C). We also conducted paraffin sections of myocardial tissue of SD rats, which subsequently analyzed using immunofluorescence techniques. The fluorescence intensity of TG2 was lower after I/R treatment than the sham group (Figure 5E; Supplementary Figure S1F). *In vitro*, H9c2 cells were cultured for 2 h with low glucose medium and a hypoxia condition, and then returned to normal culture conditions for 0 h, 2 h, 4 h, 8 h, 12 h, and 24 h, respectively. WB was employed to assess the protein expression level of TG2. The findings indicated a gradual decline in TG2 protein expression as the reperfusion time was extended (Figure 5B). We observed that the protein level of TG2 was at its lowest at the H-2 h and R-8 h time points (Figure 5B). Consequently, we chose H-2 h and R-8 h as the construction techniques for the MIRI model tailored for H9c2 cells. We detected a significant reduction in the protein expression level of TG2 in H9c2 cells following H/R exposure (2 h/8 h) compared with the control group (Figure 5D). All of these results suggested a decrease in the expression level of the TG2 protein in MIRI.

To further detect the role of GAS in the TG2 decline induced by MIRI, GAS was administered to SD rats 3 Days in advance, followed by ischemia for 45 min and reperfusion for 24 h. WB detection was performed on myocardial tissue of SD rats, and it was found that GAS also increased TG2, which was decreased at I/R (Figure 5F). This has also been verified in H9c2 cells. GAS pretreatment with concentration of 10 μM was administered 30 min before hypoxic and hypoglycemic culture of H9c2 cells. WB detection demonstrated that the pretreatment of GAS reversed H/R-mediated TG2 reduction (Figure 5G). Therefore, GAS notably suppressed the reduction in TG2 protein levels that was induced by MIRI.

### 3.6 TG2 interacts with CNPase and negatively regulates CNPase

**FIGURE 6:**
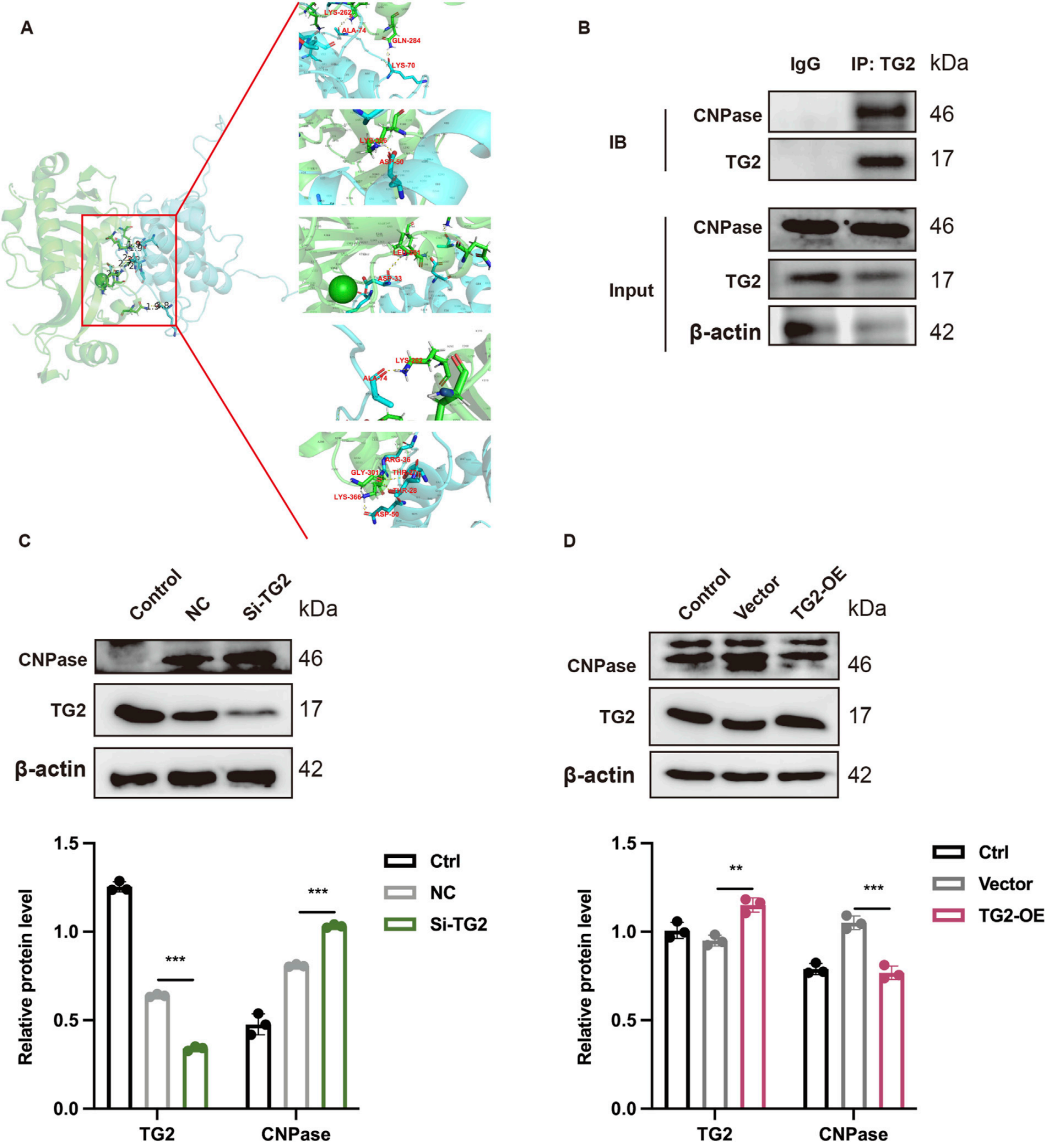
TG2 binds to CNPase and regulates the expression of CNPase. **(A)** Molecular docking diagram of TG2 and CNPase. **(B)** Co-IP technology show the combination between TG2 and CNPase. n = 3. **(C,D)** WB analysis and quantification of TG2 and CNPase when TG2 was inhibited **(C)** or increased **(D)** Results are means ± SEM of three independent experiments. n = 3. *p < 0.05, **p < 0.01, ***p < 0.001, ns, not significant.

After discovering that TG2 is a potential mechanism by which GAS improves MIRI, we delved deeper into the downstream molecular mechanism of TG2. The molecular docking site (https://gramm.compbio.ku.edu/gramm) was used to predict the relationship between TG2 and CNPase. A binding free energy of −85.58 kcal/mol was determined for the TG2-CNPase complex (Figure 6A). Following established benchmarks (Li et al., 2019), energies below −7 kcal/mol (−29.3 kJ/mol) are considered indicative of strong binding interactions, confirming significant affinity between TG2 and CNPase. Critical binding residues identified include LYS^70^, ALA^74^, ARG^36^, THR^27/28^, and ASP^33^ for TG2, with complementary residues and GLN^284^, LYS^262^, LYS^366^, GLY^301^, and LEU^281^ on CNPase. In order to further verify the interaction between TG2 and CNPase, the Co-IP technology was adopted. The Co-IP results demonstrated that the presence of TG2 could be observed in the protein sediment with anti- CNPase antibody added compared to the empty IgG group (Figure 6B). Conversely, the expression of TG2 could be detected in the protein precipitate incubated with CNPase antibody (Supplementary Figure S2B). Furthermore, the binding of TG2 and CNPase decreases as TG2 is knockdown (Supplementary Figure S2A). This finding indicates an interaction between TG2 and CNPase. We transfected H9c2 cardiocytes with either the si-RNA (Si-TG2) or the overexpression plasmid (TG2-OE) of TG2. WB analysis was then conducted to assess the protein expression level of CNPase, which was found to be significantly upregulated upon interference with TG2 expression (Figure 6C). Conversely, the expression of CNPase decreased with the increase of TG2 expression (Figure 6D). To sum up, TG2 interacts with and negatively regulates CNPase, meaning that a reduction in TG2 levels results in an increase in CNPase expression.

### 3.7 Lack of CNPase alleviates MIRI by inhibiting apoptosis

**FIGURE 7:**
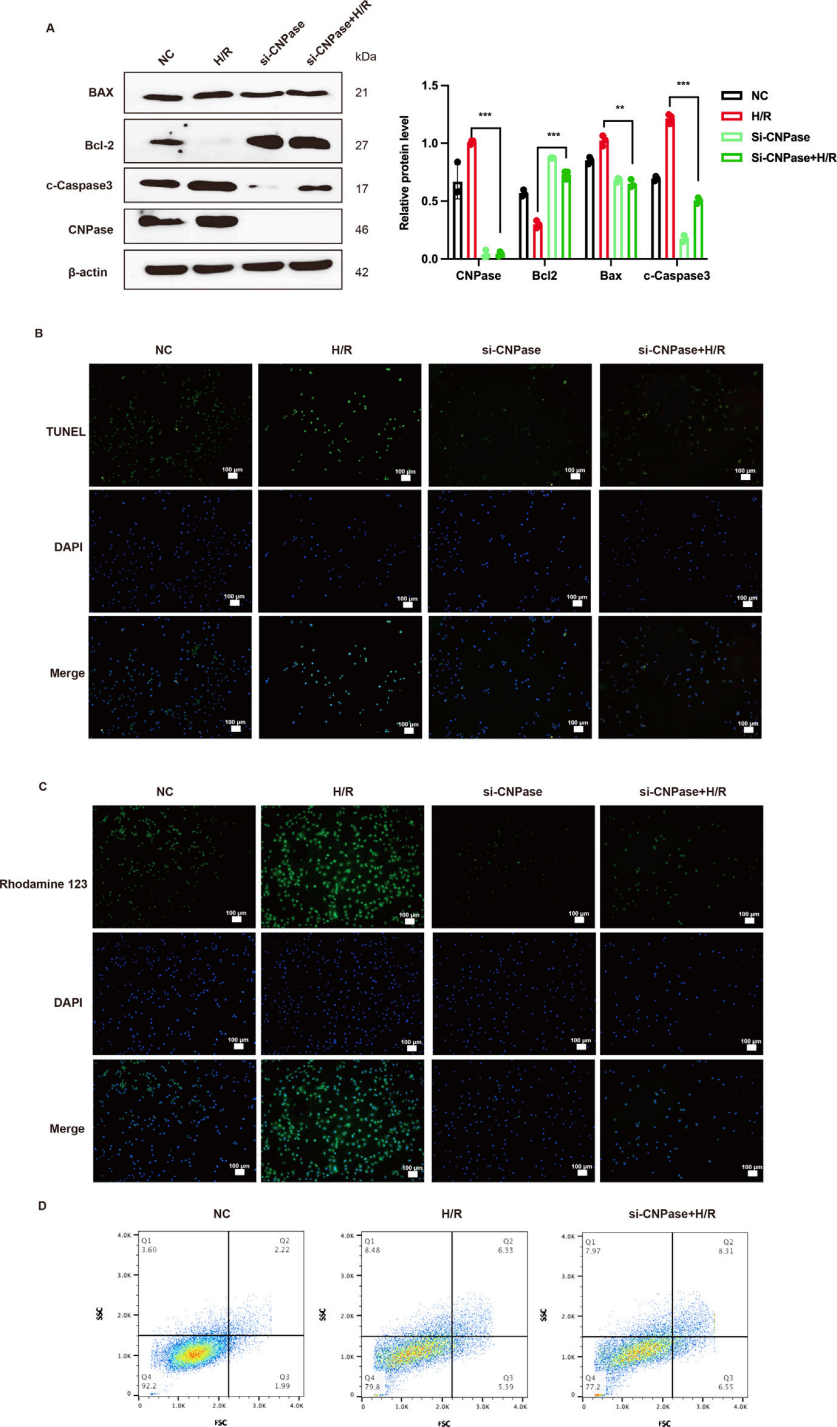
Lack of CNPase alleviates MIRI by inhibiting apoptosis. **(A)** WB analysis and quantification of CNPase, Cleaved-caspase3, Bax and Bcl-2. n = 3. **(B)** The effect of CNPase silencing on the apoptosis of H9c2 cells at H/R (2 h/8 h) was detected by Tunnel assay **(B)** and Rhodamine 123 staining **(C)** n = 3. Scale bar = 100 μm. **(D)** The effect of blocking CNPase on apoptosis of H9c2 cells was detected by Flow cytometry. n = 3. *p < 0.05, **p < 0.01, ***p < 0.001, ns, not significant.

The precise role of CNPase as a TG2’s downstream molecule during MIRI remains elusive. Therefore, we next explored the role of CNPase in MIRI. The siRNA targeting CNPase (Si-CNPase) was transfected into H9c2 cardiomyocytes. These cells were subsequently cultured under hypoxic conditions and in low-glucose medium for 2 h, followed by a return to normal culture conditions for an additional 24 h, thereby establishing an *in vitro* model of MIRI. We observed that following CNPase interference, the protein levels of cleaved-caspase3 and Bax in H/R cardiomyocytes were notably decreased compared to those in the H/R group. Conversely, the expression level of Bcl2 was markedly increased in comparison to the H/R group, suggesting that the absence of CNPase attenuated H/R-induced apoptosis (Figure 7A). The improvement effect of CNPase deletion on apoptosis was also verified by TUNNEL staining. The findings obtained were in concurrence with those derived from WB, indicating that the knockdown of CNPase mitigated the H/R-induced apoptosis (Figure 7B; Supplementary Figure S1G). Rhodamine 123 staining was mainly used to detect the apoptosis level. We used Rhodamine 123 staining to stain living cells, and the results showed that CNPase knockdown reduced the occurrence of apoptosis induced by H/R (Figure 7C; Supplementary Figure S1H). Subsequently, we transfected the CNPase siRNA into H9c2 cardiomyocytes and induced H/R in the meanwhile. Live cells were collected for PI/FITC incubation and detected by flow cytometry. The results showed that compared with the control group, the percentage of apoptotic cells in H/R group was significantly increased, while the number of apoptotic cells in H/R group after CNPase knockdown was significantly lower than that in H/R group alone (Figure 7D). In conclusion, the lack of CNPase can promote the proliferation of cardiomyocytes and improve the occurrence of apoptosis induced by MIRI.

## 4 Dicussion

MIRI constitutes a major clinical challenge during revascularization therapy for AMI patients (Heusch, 2020). Currently, no established therapy effectively prevents MIRI. Evidence indicates GAS, a bioactive compound derived from traditional Chinese medicine, mitigates MIRI pathology (Fu et al., 2018; Chen, 2024; Dong et al., 2022). However, the mechanism underlying GAS-mediated MIRI improvement remains incompletely characterized. This study demonstrates GAS’s therapeutic efficacy against MIRI through modulation of the TG2/CNPase pathway. Furthermore, we delved deeper into the mechanism by which GAS influences MIRI. GAS was found to alleviate the apoptosis induced by MIRI. Notably, CNPase was significantly increased when MIRI occurred, which was reversed by GAS pre-conditioning. TG2 physically interacts with CNPase and inversely regulated CNPase. CNPase reduction alone ameliorates MIRI-induced apoptosis, confirming its pathogenic role. Therefore, GAS confers cardioprotection by regulating the TG2/CNPase axis to suppress apoptotic signaling in MIRI (graphical abstract).

GAS, the primary bioactive constituent of the traditional Chinese herb Gastrodin elata, is a phenolic glycoside with demonstrated pharmacological activity (Liu et al., 2022). Recently, it has been discovered that GAS extends beyond its established neuropharmacological roles as a sedative and anticonvulsant to confer significant cardiovascular protection through lipid regulation, blood pressure reduction, and antithrombotic activity (Ahmad et al., 2019). Previous research demonstrated that GAS mitigated microvascular reperfusion injury induced pyrodeath by modulating the NLRP3/caspase-1 pathway (Sun et al., 2019). GAS has also been shown to attenuate MIRI by improving mitochondrial phagocytosis through the PINK1/Parkin pathway (Chen, 2024). To further investigate the role of GAS in MIRI, SD rats received intraperitoneal GAS for three consecutive days, followed by I/R surgery. GAS pretreatment significantly reduced the size of heart infarctions caused by I/R compared with the MIRI group, consistent with prior findings (Chen, 2024). In addition, the isolated heart perfusion technique showed that GAS pretreatment ameliorated MIRI-induced cardiac dysfunction in SD rats. Specifically, GAS pretreatment enhanced cardiac systolic function and elevated myocardial oxygen consumption in SD rats. These findings extend prior observations of GAS-mediated cardioprotection (Sun et al., 2019) and align with recent mechanistic insights (Chen, 2024).

While GAS’s efficacy against MIRI is confirmed, its precise mechanism still needs to be further dug out. TG2 is widely expressed in smooth muscle cells as an actin-binding protein (Rao et al., 2015). It was found that TG2 not only promotes relaxation of airway smooth muscle cells (Yin et al., 2018), but also regulates the cytoskeleton of endothelial cells (Xiao et al., 2012). Studies have shown that TG2, as a membrane protein, increases the stability of actin in immune synapses (Goodman et al., 2003; Na et al., 2015). Szelenberger et al. found that the protein and mRNA of TG2 was changed in the platelet proteome of patients with Acute coronary syndrome (ACS) (Szelenberger et al., 2022). Notably, the role of TG2 in cardiomyocytes remains unreported. To investigate its function in MIRI, we constructed *in vivo* H/R and *in vitro* I/R reperfusion time gradient models, and detected the protein expression level of TG2. We found that TG2 decreased with prolonged reperfusion time. Furthermore, our findings revealed that, *in vitro*, the expression level of TG2 reached its nadir at 2 h of oxygen-glucose deprivation followed by 12 h of reoxygenation. The lowest expression level of TG2 was observed at 45 min of ischemia and 24 h of reperfusion *in vivo*. Similarly, the IF results confirmed the reduction of TG2 at MIRI. These results suggest cardiomyocyte TG2 depletion may derive MIRI pathogenesis. This contrasts with reported TG2 upregulation in hypoxic cancer cells, where it activates the insulin-like growth factor 1 receptor beta (IGF1Rβ)/PI3K/AKT signaling pathway, which in turn facilitates the process of epithelial-mesenchymal transition (EMT) (Kim et al., 2018). The differential regulation likely reflects tissue-specific signaling. Crucially, GAS treatment reversed MIRI-induced TG2 downregulation, indicating TG2 restoration mediates GAS cardioprotection.

Further investigation TG2’s mechanism revealed that TG2 can interact with CNPase, and the results of Co-IP suggest that the two combine with each other. In recent years, the crucial role of CNPase in cardiovascular disease has also been confirmed (Wang et al., 2021). Tan et al. have shown that CNPase attenuates myocardial hypertrophy through enhanced mitochondrial energetics, positioning it as a therapeutic target in heart failure (Tan et al., 2021). Additionally, elevated CNPase in acute heart failure confers myocardial protection (Odinokova et al., 2018). In line with previous research, our findings revealed that both myocardial ischemia and MIRI resulted in an upregulation of CNPase expression. However, CNPase expression was notably downregulated during myocardial reperfusion compared to myocardial ischemia alone. Previous research has indicated that the elevated expression of CNPase in heart failure exerts a protective influence on the myocardium. This protective effect is primarily ascribed to CNPase’s ability to decrease the opening of mPTP, thereby inhibiting the release of mitochondrial apoptotic proteins (Odinokova et al., 2018). Our CNPase knockdown experiments paradoxically reduced MIRI-induced cardiomyocyte apoptosis. This is different from the results of previous studies in which CNPase alleviates apoptosis, which may be caused by different study objects, or because we studied total proteins and did not separate mitochondrial proteins for study. Notably, GAS preconditioning reversed MIRI-mediated increased expression of CNPase. In conclusion, GAS improves MIRI by regulating TG2/CNPase signaling.

Interestingly, this study provides the first evidence that TG2 binds to and negatively regulates CNPase, extending TG2’s known as an actin-binding protein (Rao et al., 2015). Moreover, prior research demonstrates that CNPase directly interacts with actin cytoskeletal networks (Markusson et al., 2024). While the interaction of TG2 with CNPase was previously undocumented, this study demonstrates their physical association and reveals that TG2 deficiency upregulates CNPase expression.

Our findings align with reports on plant-derived cardioprotectants, yet demonstrate distinct mechanistic advantages. Whereas salvianolic acid B attenuates MIRI via modulating SIRT3-mediated crosstalk between mitochondrial ROS and NLRP3 (Wei et al., 2025), GAS uniquely targets TG2/CNPase-mediated apoptosis. Compared to clinical-phase compounds like TRO40303 (mitochondrial permeability inhibitor) which showed limited efficacy in Phase II (Hansson et al., 2015), GAS offers multi-faceted protection. Crucially, no prior studies report TG2-CNPase physical interaction–a novel contribution to MIRI therapeutics.

One limitation of this study is that it solely focuses on the protein-protein interaction between TG2 and CNPase, without conducting an in-depth analysis of the interaction type to elucidate the underlying mechanism of their interplay. Although the precise mechanism by which TG2 negatively regulates CNPase remains to be fully elucidated, potential pathways include direct protein-protein interaction-induced functional suppression or post-translational modifications such as ubiquitination. Further studies are needed to validate this hypothesis in the context of CNPase regulation. While this work elucidates the TG2/CNPase-mediated mechanism underlying GAS’s cardioprotective effects against MIRI, the therapeutic translation potential remains unexplored, particularly regarding optimal dosing regimens, pharmacokinetics, and safety profiles of GAS in clinical MIRI contexts.

## 5 Conclusion

In summary, our research indicates that GAS preconditioning exerts a protective effect on MIRI by improving apoptosis via the TG2/CNPase signaling pathway. Our study has uncovered the relationship between TG2 and CNPase, offering not only a novel therapeutic target for the treatment of MIRI with GAS but also a solid theoretical foundation for exploring new diagnostic avenues and therapeutic approaches for MIRI.

## Data Availability

The original contributions presented in the study are included in the article/Supplementary Material, further inquiries can be directed to the corresponding author.
