# Supplementary material for: Gastrodin reduces myocardial ischemia/reperfusion injury via transgelin2/CNPase-mediated apoptosis regulation
Source: Front Pharmacol. 2025 Jul 14;16:1604408. doi: 10.3389/fphar.2025.1604408 (PMC12301622; doi:10.3389/fphar.2025.1604408)
Supplement: Supplementary file 1 [file Supplementaryfile1.docx]

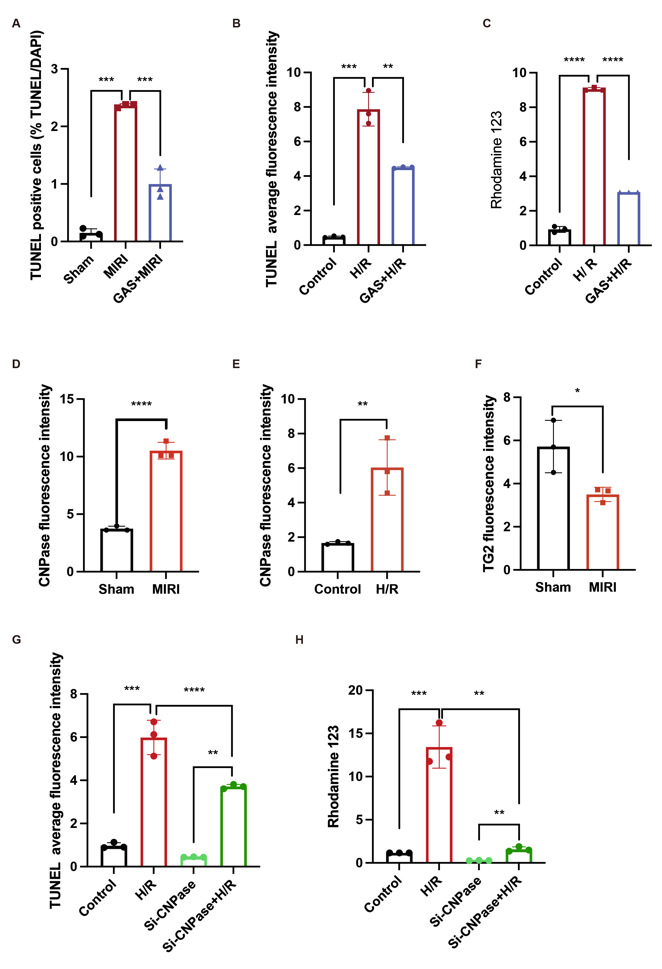


**Supplementary figure 1. Statistical graph of fluorescence staining.**


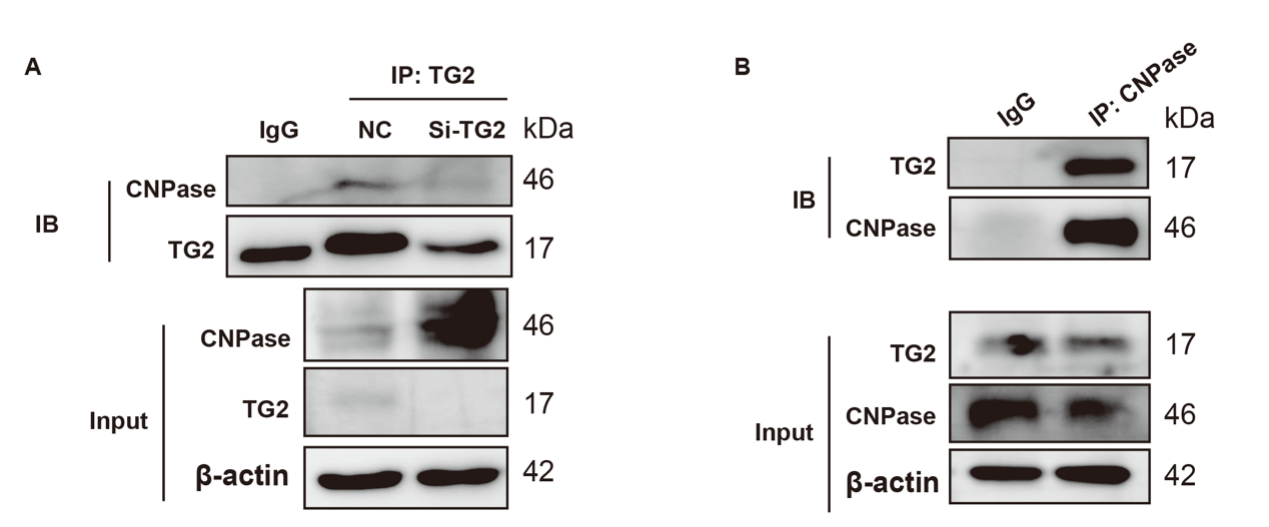


**Supplementary figure 2. Co-IP verification of TG2 and CNPase combination.** A. Co-IP detects the binding of TG2 to CNPase after interfering with TG2. B. Reverse Co-IP verification of TG2 and CNPase.


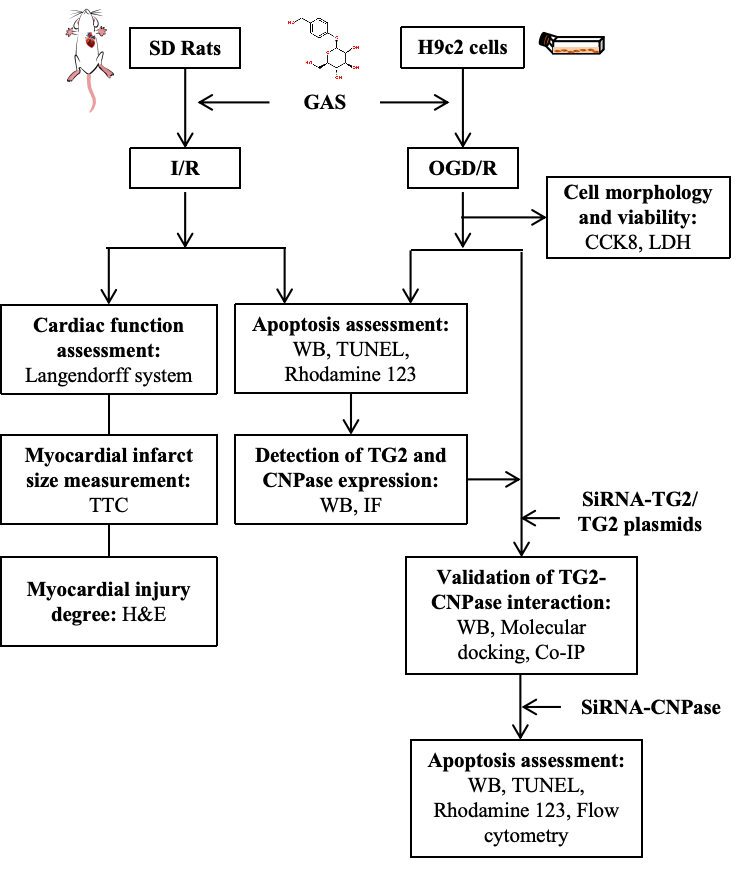


**Supplementary figure 3. The full text technical roadmap.**
